# An efficient method for the sanitary vitrification of bovine oocytes in straws

**DOI:** 10.1186/2049-1891-5-19

**Published:** 2014-04-11

**Authors:** Yanhua Zhou, Xiangwei Fu, Guangbin Zhou, Baoyu Jia, Yi Fang, Yunpeng Hou, Shien Zhu

**Affiliations:** 1 National Engineering Laboratory for Animal Breeding, Key Laboratory of Animal genetics, Breeding and Reproduction, Ministry of Agriculture, College of Animal Science and Technology, China Agricultural University, Beijing 100193, P.R. China; 2 State Key Laboratory for Agrobiotechnology, College of Biological Sciences, China Agricultural University, Beijing 100193, P.R. China; 3 Institute of Animal Genetics and Breeding, College of Animal Science and Technology, Sichuan Agricultural University (Chengdu Campus), Wenjiang 611130, P.R. China

**Keywords:** Bovine, Cryopreservation, Oocytes, Straw, Vitrification

## Abstract

**Background:**

At present, vitrification has been widely applied to humans, mice and farm animals. To improve the efficiency of vitrification in straw, bovine oocytes were used to test a new two-step vitrification method in this study.

**Results:**

When *in vitro* matured oocytes were exposed to 20% ethylene glycol (EG20) for 5 min and 40% ethylene glycol (EG40) for 30 s, followed by treatment with 30% glycerol (Gly30), Gly40 or Gly50, a volume expansion was observed in Gly30 and Gly40 but not Gly50. This indicates that the intracellular osmotic pressure after a 30 s differs between EG40 and ranged between Gly40 (approximately 5.6 mol/L) and Gly50 (approximately 7.0 mol/L). Since oocytes are in EG40 just for only a short period of time (30 s) and at a lower temperature (4°C), we hypothesize that the main function of this step in to induce dehydration. Based on these results, we omitted the EG40 step, before oocytes were pretreated in EG20 for 5 min, exposed to pre-cooled (4°C) Gly50, for 30 s, and then dipped into liquid nitrogen. After warming, 81.1% of the oocytes survived, and the surviving oocytes developed into cleavage stage embryos (63.5%) or blastocysts (20.0%) after parthenogenetic activation.

**Conclusions:**

These results demonstrate that in a two-step vitrification procedure, the permeability effect in the second step is not necessary. It is possible that the second step is only required to provide adequate osmotic pressure to condense the intracellular concentration of CPAs to a level required for successful vitrification.

## Background

Vitrification is the rapid cooling of cells in liquid medium in the absence of ice crystal formation. Vitrification can be achieved when the intracellular concentration of cryoprotective agents (CPAs) is higher than 6 mol/L [1]. The benefits of a two-step vitrification method are that it allows establishment of a relatively complete equilibrium while reducing exposure of the oocyte to potential toxic effects of CPAs. Previously, oocytes or embryos were first exposed to non-vitrifying solutions containing permeating CPAs [2,3]. Next, the oocytes were exposed for a short time (45–60 s) to a vitrifying solution (VS) containing high concentrations of penetrating (4.8–6.4 mol/L) and non-penetrating (0.5–0.75 mol/L) CPAs before being plunged into liquid nitrogen (LN2) [2-4].

Since the first successful vitrification of mouse embryos by Rall and Fahy in 1985 [1], this method has been used widely for oocyte and embryo cryopreservation. Numerous research articles have focused on CPA permeability and the rate at which it enters cells [5,6]. Other studies have investigated incubation times in both the pretreatment and vitrification solutions and found that the temperature used during the handling procedure is also important for successful vitrification [7-9]. The open-pulled straw (OPS) method originally described by Vajta and colleagues, allows for faster heat transfer between the solution and the environment, achieving cooling/warming rates on the order of 20,000°C/min [10]. In 1999, when Le Gal and Massip compared three approaches (standard 0.25-mL straw, OPS, and Microdrop) for cooling a vitrification solution containing bovine oocytes, the highest cleavage rate was achieved with the traditional straw [11]. Dinnyés and colleagues [12] described the use of solid-surface vitrification (SSV). In 2004, an early report using cryotops for bovine oocyte vitrification was published [13]. These variations make the vitrification method seem difficult to master which has limited the application of this technology in the field of reproductive biology.

Cells react to changes in extracellular osmolarity by altering their volume. Cells exposed to hypotonic or hypertonic solutions initially react either by swelling (hypotonic solutions) or shrinking (hypertonic solutions) due to water exchange but later recover as permeant solutes equilibrate across the cell membrane [4,5,14,15]. Vanderzwalmen et al. [3,4] estimated the final intracellular concentration of cryoprotectant (ICCP) after incubation in vitrification solutions by exposing cells to sucrose solutions with defined molarities. The ICCP was calculated from the sucrose concentration that produced no change in cell volume, i.e., when intra- and extracellular osmolarities were equivalent [4].

In 1977, Whittingham successfully cryopreserved mouse oocytes [16]. Bovine oocytes were also vitrified and remained viable for offspring production after *in vitro* fertilization and embryo transplantation [17,18]. vitrified buffalo oocytes with 51.1% glycerol via the straw method, obtaining a maturation rate of 23.5% after thawing [18]. When glycerol was used with EG, which increased permeability of the cell membrane during oocyte vitrification, and maturation rates of 30 s exposure groups did not differ from those of controls [19]. Additionally, the OPS (open pulled straw) method results in a better survival rate during cryopreservation than the straw method [20]. However, unlike other methods, the straw method is safer for oocyte vitrification because the oocytes are free of bacterial contamination due to a lack of direct contact with liquid nitrogen.

In our experiments glycerol was used as an extracellular measure for ICCP. In the first part of this study, bovine oocytes were used to test changes in intracellular cryoprotectant concentration during a widely used two-step vitrification method. Oocytes were pretreated with 20% EG (EG20) for 5 min, transferred to pre-cooled (4°C) 40% EG (EG40) for 30 s, then treated with pre-cooled glycerol either at 30% (Gly30), 40% (Gly40) or 50% (Gly50) concentration. The intracellular EG molarity was then determined from the extracellular glycerol molarity. In the second part of the experiment, oocytes were pretreated with EG20 for 5 min, transferred directly to pre-cooled (4°C) 50% glycerol (Gly50) for 30 s, and then plunged directly into liquid nitrogen for cryopreservation in an insemination straw. Vitrified-warmed oocytes were parthenogenetically activated and cultured *in vitro* to assess viability.

In this study, experiments were designed to improve the efficacy of vitrification in straws. To optimize the ideal CPA treatment for this two-step vitrification method, different cryoprotectants (EG and Gly) were used in each step, which differs from methods reported previously. It has been reported that the permeability of glycerol is relatively low [14]. The present experiments examined whether CPA permeability during the second step is a key factor for vitrification. We investigated the possibility that the second equilibration step provides a high osmotic pressure increase intracellular CPA to a level required for successful vitrification.

## Methods

The Institution Animal Care and Use Committee at China Agricultural University (Beijing, China) reviewed and approved the protocols used in this study. All chemicals and media were purchased from Sigma Chemical Co. (St. Louis, MO, USA) unless otherwise indicated.

### Solution preparation

Modified phosphate-buffered saline (mPBS) was prepared by adding 10% (v/v) fetal bovine serum (FBS, Gibco), 0.3% (w/v) BSA and 50 mg/mL gentamycin to Dulbecco’s phosphate-buffered saline (DPBS, Gibco).

EG20 was prepared by adding 20% (v/v) ethylene glycol to mPBS;

EG40 was prepared by adding 40% (v/v) ethylene glycol to mPBS;

Gly30 was prepared by adding 30% (v/v) glycerol to mPBS;

Gly40 was prepared by adding 40% (v/v) glycerol to mPBS;

Gly50 was prepared by adding 50% (v/v) glycerol to mPBS;

Dilution medium was 0.5 mol/L sucrose in mPBS.

### Oocyte collection and *in vitro* maturation

Bovine (*Bos taurus*, 3 to 6 yr of age) ovaries were transported from the abattoir to the laboratory in a physiological saline solution at 26°C to 30°C within 2 h of slaughter. Antral follicles (2 mm to 8 mm in diameter) were manually aspirated using an 18-gauge needle attached to a 10 mL syringe. Oocytes with at least four layers of compact cumulus cells (COCs) were selected for *in vitro* maturation (IVM). Oocytes were washed three times in HEPES-buffered TCM-199 medium and then washed twice in NaHCO_3_-buffered TCM-199. Fifty COCs were transferred to 0.75 mL maturation medium (M199 with 10 mg/mL oFSH [Ovagen, Auckland, New Zealand], 10 mg/mL oLH [Ovagen], 1 mg/mL estradiol [Ovagen] and 10% fetal bovine serum [FBS; Gibco]) in 4-well plates (Nunclon). The COCs were cultured for 22 h at 38.5°C in a humidified atmosphere with 5% CO_2_. Oocytes were denuded after 22 h maturation by repeated pipetting with a 200 μL pipette for approximately 1 min in 38°C 0.1% w/v. hyaluronidase. Cumulus-free oocytes with the first polar body were selected and randomly allocated to experimental groups.

### Oocyte volume

Oocyte volumes were determined using established methods [14] with modifications. Oocytes were fixed in a 5 μL mPBS drop using a holding needle (outer diameter 50 μm; inner diameter 30 μm) attached to an Olympus inverted microscope, 200 μL of EG20 was then flushed onto the drop. The drop of EG20 (~200 μL) was aspirated 5 min later with a transferpettor. In the second step, 500 μL of pre-cooled (4°C) EG40 (experiment 1-a) or Gly50 (experiment 1-e) was flushed into the drop. For Experiments 1-b, c and d, 200 μL pre-cooled solution (EG40) was flushed into the drop. EG40 was aspirated off the oocyte after 30 s, and then 500 μL of pre-cooled (4°C) glycerol solution (either of Gly30, Gly40 or Gly50) was flushed into the drop.

The entire procedure was video recorded using a CCD camera on an inverted microscope. Screenshots of the video recording were taken at the desired times. Cross-sectional areas of the oocytes were calculated using EZ-C1 3.00 Free Viewer software. The relative change in volume was determined according to a previously pubulished method [10]. Briefly, the oocyte area relative to that in isotonic mPBS medium was calculated and converted into a relative volume (considered as 1 V). The volume was assumed to change proportionally, and the equation V = S^3/2^ was used, where S is relative cross-sectional area and V is the relative volume. For each treatment, 5 oocytes were examined.

### Oocyte vitrification and warming

**Figure 1:**
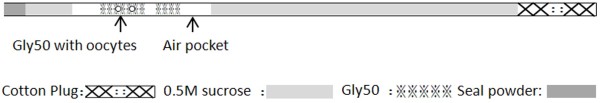
Cubing protocol: the sucrose solution in the plug end occupies 5.0 cm, the section in which the oocytes are placed occupies 1.2 cm and a small volume of Gly50 lies to the right of the oocytes.

Oocytes were vitrified by a two-step method as previously reported, with modifications [9,21,22]. Briefly, oocytes were placed in EG20 for 5 min at 25°C. The oocytes were then transferred to pre-cooled Gly50 for 30 s, pipetted into sections of an insemination straw (250 μL, IMV, L’Aigle, France), as shown in Figure 1. and then straws were sealed with seal powder and plunged into liquid nitrogen. Two oocytes were loaded into each straw.

After one week of storage in liquid nitrogen, the straws were plunged into 25°C water for 10 s. As the crystallized sucrose solutions in the straw melted, the straws were removed from the water and quickly wiped dry. The straws were then held at the sealed end and shaken three times by hand to mix the vitrification solution and the sucrose solution. Subsequently, the seals of the straw were removed and the oocytes were expelled from the straw into a dry culture dish. Oocytes were put into fresh 0.5 mol/L sucrose for 5 min and then washed in two other mPBS dishes for 5 min each.

After a 30 min recovery in mPBS, the oocytes were assessed for survival. Surviving oocytes were those with regular, spherical shapes that were not lysed, shrunken, swollen or blackened. The surviving oocytes were parthenogenetically activated and cultured *in vitro*.

### Parthenogenetic activation

Oocytes were washed three times in HEPES-buffered TCM-199 with 10% FBS (H199) and then activated as follows: (1) incubation for 5 min in 7% ethanol in IVM medium at room temperature and (2) cultured for 4 h in 2 mmol/L 6-DMAP in culture medium. Fifteen oocytes were transferred to 60 μL Charles Rosenkran’s 1 medium [23] with BSA (3 mg/mL, Sigma A3311) covered with mineral oil (Sigma M8410) in a 35 mm × 35 mm Nunclon dish and cultured in an incubator (38.5°C with 5% CO_2_ in air) for up to 48 h before determining the rates of activation and cleavage. Cleaved embryos were cultured for an additional 5 d in Charles Rosenkran’s 1 medium with 5% FBS.

### Experimental design

Experiment 1. In this section oocytes were randomly allocated to five experimental groups, and each experiment was repeated five times.

(a) Oocytes were incubated in EG20 for 5 min followed by addition of pre-cooled (4°C) EG40. Oocytes → EG20 (25°C, 5 min) → EG40 (4°C, 4 min).

(b) Oocytes were incubated in EG20 for 5 min, pre-cooled (4°C) EG40 for 30 s, and then incubated in pre-cooled (4°C) Gly30 for 3 min. Oocytes → EG20 (25°C, 5 min) → EG40 (4°C, 30 s) → Gly30 (4°C, 3 min).

(c) Oocytes were incubated in EG20 for 5 min, pre-cooled (4°C) EG40 for 30 s, and then pre-cooled (4°C) Gly40 for 3 min. Oocytes → EG20 (25°C, 5 min) → EG40 (4°C, 30 s) → Gly40 (4°C, 3 min).

(d) Oocytes were incubated in EG20 for 5 min, pre-cooled (4°C) EG40 for 30 s, and then pre-cooled (4°C) Gly50 for 3 min. Oocytes → EG20 (25°C, 5 min) → EG40 (4°C, 30 s) → Gly50 (4°C, 3 min).

(e) Oocytes were incubated in EG20 for 5 min and then pre-cooled (4°C) Gly50 for 4 min. Oocytes → EG20 (25°C, 5 min) → Gly50 (4°C, 4 min).

The volume changes of oocytes during all of these procedures were analyzed and used to generate a curve diagram over time.

Experiment 2. Development of cryopreserved oocytes after parthenogenetic activation.

Cumulus-free oocytes with the first polar body and normal morphology were selected and allocated randomly to the following experimental groups:

(1) Control group: Oocytes without CPA treatment or vitrification were cultured after parthenogenetic activation.

(2) Toxicity group: Oocytes were exposed to the same solutions as the vitrification group but were not plunged into liquid nitrogen. These oocytes were diluted and parthenogenetically activated according to the procedure used for the vitrification group.

(3) Vitrification group: based on the results of experiment 1, oocytes were pre-treated in EG20 (25°C) for 5 min and then transferred to Gly50 (4°C) for 30 s before being plunged into liquid nitrogen.

### Statistical analysis

Embryos development experiments were repeated three times. The percentage data were subjected to arcsine transformation before statistical analysis. The data were analyzed by one-way ANOVA combined with the LSD test. *P* <0.05 was considered statistically significant.

## Results

**Figure 2:**
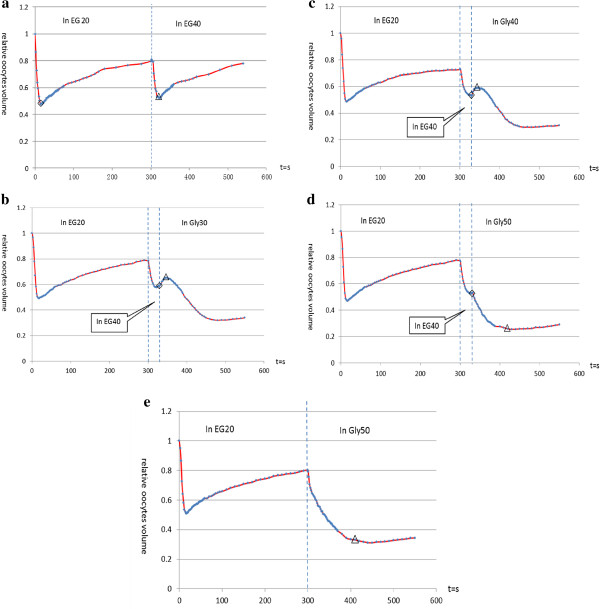
**Volume changes within bovine MII-stage oocytes during different CPA treatments. a)** oocytes pre-treated with EG20 for 5 min and then transferred to pre-cooled EG40; **b)** oocytes pre-treated with EG20 for 5 min, transferred to pre-cooled (4°C) EG40 for 30 s, and then flushed with pre-cooled (4°C) Gly30; **c)** oocytes pre-treated with EG20 for 5 min, transferred to pre-cooled (4°C) EG40 for 30 s, and then flushed with pre-cooled (4°C) Gly40; **d)** oocytes pre-treated with EG20 for 5 min, transferred to pre-cooled (4°C) EG40 for 30 s, and then flushed with pre-cooled (4°C) Gly50; **e)** oocytes after pre-treatment with EG20 for 5 min and then transfer to pre-cooled Gly50.

Experiment 1.Oocytes volume changes for five different protocols:

(a) Oocytes → EG20 (25°C, 5 min) → EG40 (4°C, 4 min). When exposed to EG20, the oocytes shrank to 0.48 V (◇ in Figure 2a) in 20 s and then swelled slowly to 0.80 V after 5 min of exposure. When the oocytes were flushed with pre-cooled EG40, they shrank to 0.52 V (△ in Figure 2) and then gradually swelled again.

(b) Oocytes → EG20 (25°C, 5 min) → EG40 (4°C, 30 s) → Gly30 (4°C, 3 min). At the end of the 30 s exposure to EG40, the oocytes shrank to 0.59 V (◇ in Figure 2b). Subsequently, pre-cooled Gly30 was flushed over the oocytes and an expansion in volume to 0.65 V was observed (from ◇ to △, as shown in Figure 2b), indicating a higher intracellular osmotic pressure as compared to the extracellular pressure. After a 25-s exposure to Gly30, the oocytes began to gradually shrink in volume.

(c) Oocytes → EG20 (25°C, 5 min) → EG40 (4°C, 30 s) → Gly40 (4°C, 3 min). As shown in Figure 2c (from ◇ to △), the oocytes swelled from 0.53 V to 0.59 V, indicating that the intracellular osmotic pressure after treatment was higher than the extracellular osmotic pressure generated by Gly40.

(d) Oocytes → EG20 (25°C, 5 min) → EG40 (4°C, 30 s) → Gly50 (4°C, 3 min). As shown in Figure 2d, when the oocytes were flushed with pre-cooled Gly50 followed by a 5 min treatment with EG20 and a 30 s treatment with EG40, no expansion was observed. After immersion in Gly50, the oocytes began to shrink, and within 60 s, the oocytes gradually reached a minimum volume (from ◇ to △ in Figure 2d). This result indicated that the intracellular osmotic pressure was not higher than the extracellular osmotic pressure exerted by Gly50.

(e) Oocytes → EG20 (25°C, 5 min) → Gly50 (4°C, 4 min). In this experiment, oocytes were pretreated with EG20 and then flushed with pre-cooled Gly50. Volume changes are shown in Figure 2e. The oocytes shrank quickly in Gly50 after EG20 pretreatment. The oocytes reached a minimum volume (△ part in Figure 2e) within approximately 100 s.

Experiment 2. Development of cryopreserved oocytes after parthenogenetic activation

As shown in Table 1, after vitrification, warming and parthenogenetic activation, surviving bovine oocytes develop to the cleavage stage embryos and blastocysts. After this vitrification protocol, 81.1% oocytes survived and 63.5% of them cleaved after parthenogenetic activation. Finally, we observed a 20.0% blastocyst rate. There was no difference in rates of blastocyst development between the control and toxicity groups (38.6% *vs* 36.0%, *P* > 0.05). However, after oocyte vitrification, the rates of blastocyst development decreased (*P* < 0.05).

**Table 1:** Effects of oocyte vitrification on embryo development after parthenogenetic activation

**Group**	**No. oocytes**	**Survival rate**	**Cleavage rate**	**Blastocyst rate**
Control	132	100% ± 0 (132/132)^a^	84.21% ± 2% (111/132)^a^	38.62% ± 0.75% (51/132)^a^
Toxicity	103	93.22% ± 1.56% (96/103)^b^	76.74% ± 2.27% (79/96)^b^	35.93% ± 1.47% (37/96)^a^
Vitrified	105	81.08% ± 2.86% (89/105)^c^	63.49% ± 2.1% (66/105)^c^	19.96% ± 1.06% (21/105)^b^

Different superscripts (a, b, c) in the same column represent a significant difference (*P* < 0.05). Percentage data are presented as mean ± SEM.

## Discussion

There are few reports that analyze vitrification of bovine metaphase oocytes by the straw method. In the present study, we achieved cleavage (63.5%) and blastocyst development (20.0%) after parthenogenetic activation of vitrified-warmed bovine oocytes similar to that from oocytes vitrified by the open-pulled straw method (57.0% cleavage and 23.0% blastocyst development, respectively) [24]. However, the straw method has an advantage for bovine oocyte vitrification, because oocytes do not directly contact the liquid nitrogen and thus potential bacterial contamination is avoided.

Most two-step vitrification methods are similar, with some differences in exposure time or CPA combination. In fact, exposure times greatly influence the outcome of the vitrification method [2,7-9,22]. It has been reported that when bovine blastocysts are exposed to EFS40 from 1 to 3 min, the survival rate drops from 77.0% to 7.0% [8]. Campos-Chillon tested a range of pretreatment times (from 1 to 3 min) in 3.5 mol/L ethylene glycol on bovine morulae and found that a 1-min exposure was ideal [7]. Fujihira found a significant relationship (*P* < 0.05) between the rate of development of morphologically normal oocytes after vitrification and equilibration time in pigs [25]. Others used much longer exposure times (10-20 min) for oocytes [19] or embryos [21,26] before vitrification. Despite these variations, the ultimate goal of these procedures was to determine proper intracellular concentrations of CPA for successful vitrification of oocytes.

In the present study, bovine oocytes exposed to EG20 for 5 min and then transferred to EG40 shrank to 0.52 V before gradually regaining volume. Furthermore, mouse [5] and bovine [15] oocytes exhibited ideal osmotic responses when their volumes were analyzed using the Boyle-van’t Hoff relationship. Here, cells stopped shrinking at 0.52 V, which can be inferred as the state of equilibrium between intra- and extracellular osmotic pressures resulting from exposure to hypotonic (swelling) or hypertonic (shrinking) solutions [4]. In subsequent experiments, oocytes were sequentially exposed to EG20 (5 min) and EG40 (30 s) followed by treatment either with Gly30 and Gly40 or Gly50. Volume expansion was observed in Gly30 and Gly40, suggesting that the intracellular osmotic pressure was higher than that produced by Gly40. However, volume expansion did not occur when the oocytes were flushed with Gly50, suggesting that the extracellular osmotic pressure was higher in Gly50 than the intracellular osmotic pressure.

The permeability of CPAs is strongly decreased at low temperatures [15,27]; therefore, during a short exposure of oocytes (30 s) to pre-cooled (4°C) EG40 in the second equilibrium step, the intra- and extracellular EG levels changed minimally. As a result, we omitted the EG40 step and modified the second equilibrium step by exposing oocytes to EG20 for 5 min and to pre-cooled Gly50 for 30 s before plunging into liquid nitrogen. As shown in Figure 2e, the oocytes shrank to a minimum volume in approximately 100 s, and this shrinking would result in concentration of intracellular EG during this period. It has been reported by Jin [28] that vitrification solutions with higher osmotic pressure could facilitate intracellular vitrification, yielding better results. In Experiment 2, oocytes were vitrified after treatment with pre-cooled (4°C) Gly50 for 30 s. After warming, 81.1% of the oocytes survived, and the surviving oocytes developed into cleavage stage embryos (63.5%) and blastocysts (20.0%) after parthenogenetic activation. As long as the CPA concentration higher than EFS30 (which corresponds to PB1 medium containing 30% (v/v ethylene glycol, 21% (w/v) Ficoll 70, and 0.35 mol/L sucrose) oocytes will not devitrify during warming [29]. The high survival of oocytes here which indicates that the intra- and extracellular CPAs were vitrified during both the freezing and warming procedures.

In this study, we did not compare oocyte survival and development in the absence and presence of Gly50 (after EG20 (5 min) > EG40 (30 s)). We believed that treatment with Gly50 would yield better results, as it has been reported that solutions containing 40% ethylene glycol remain transparent when plunged into liquid nitrogen but crystallize during warming [29]. Although we can not rule out the possibility that EG leaves the oocytes under high osmotic pressure, the concentration of EG remaining is sufficient to achieve vitrification according to our results in Table 1.

## Conclusion

Results from these experiments provide clear evidence that during the two-step equilibrium before vitrification, if proper pretreatment (EG20 for 5 min) was taken, the permeability of CPA into oocytes is unnecessary during the second equilibration step. What is efficient is a vitrification solution (Gly50) with high osmotic pressure only during the second equilibrium period to concentrate the intracellular CPAs adequately to facilitate intracellular vitrification.

## Abbreviations

EG20: 20% v/v ethylene glycol in mPBS; EG40: 40% v/v ethylene glycol in mPBS; Gly30: 30% v/v glycerol in mPBS; Gly40: 40% v/v glycerol in mPBS; Gly50: 50% v/v glycerol in mPBS; EFS30: PB1 medium containing 30% v/v ethylene glycol, 21% (w/v) Ficoll 70, and 0.35 mol/L sucrose; CPA: Cryoprotective agents; ICCP: Intracellular concentration of cryoprotectant; VS: Vitrification solution; V: Volume; S: Sucrose; Min: Minute; Sec: Second.

## Competing interests

The authors declare that they have no competing interests.

## Authors’ contributions

ZYH designed the study, conducted the experiments and analyses and wrote the manuscript. ZSE collaborated in the design of the study and analysis and oversaw the work of laboratory staff. FXW collaborated in the design of the study and the analysis and helped write the manuscript. ZGB collaborated in the design of the study and reviewed the manuscript. JBY selected the assays to measure cross-sectional area and assisted in the interpretation of results. FY helped with the culture of oocytes and early embryos. HYP collaborated in the interpretation of results and writing of the manuscript. All authors read and approved the final manuscript.

## Authors’ information

Yanhua Zhou is Ph.D candidate of College of Animal Science and Technology, China Agricultural University, Shien Zhu is professor of College of Animal Science and Technology, China Agricultural University. Xiangwei Fu is associate professor of College of Animal Science and Technology, China Agricultural University, Yunpeng Hou is associate professor of State Key Laboratory for Agrobiotechnology, College of Biological Sciences, China Agricultural University, Guangbin Zhou is professor of College of Animal Science and Technology, Sichuan Agricultural University (Chengdu Campus). Baoyu Jia and Yi Fang are Ph.D candidate of College of Animal Science and Technology, China Agricultural University.
